# Low prevalence of syphilis infection among key populations in Togo in 2017: a national cross-sectional survey

**DOI:** 10.1186/s13690-019-0365-x

**Published:** 2019-09-05

**Authors:** Didier K. EKOUEVI, Alexandra M. BITTY-ANDERSON, Fifonsi A. GBEASOR-KOMLANVI, Yao R. KONU, Essèboè K. SEWU, Mounerou SALOU, Claver A. DAGNRA

**Affiliations:** 1 Faculté des Sciences de la Santé, Département de Santé Publique, Université de Lomé, Lomé, Togo; 2 Université de Bordeaux, Institut de Santé Publique Epidémiologie Développement (ISPED), Bordeaux, France; 3 Programme PACCI – Site ANRS Côte d’Ivoire, CHU de Treichville, Abidjan, Côte d’Ivoire; 4 INSERM U1219 Bordeaux Population Health Research, ISPED, Université de Bordeaux, Bordeaux, France; 5 Centre Africain de Recherche en Epidémiologie et en Santé Publique (CARESP), Lomé, Togo; 6 Laboratoire de Biologie Moléculaire, Département des Sciences Fondamentales, Université de Lomé, Lomé, Togo; 7 Programme National de Lutte contre le VIH/Sida et les Infections Sexuellement Transmissibles (PNLS/IST), Lomé, Togo

**Keywords:** Syphilis, HIV, Prevalence, Key populations, Sub-Saharan Africa

## Abstract

**Background:**

The World Health Organisation (WHO) recommends the screening of syphilis among populations highly exposed to HIV. However, data on the prevalence of syphilis in these populations are scarce in Togo. This study aimed at estimating the prevalence of syphilis among males who have sex with males (MSM), female sex workers (FSW) and drug users (DU) in Togo.

**Methods:**

A cross-sectional bio-behavioral study was conducted in August and September 2017 using a respondent-driven sampling (RDS) method in eight major cities in Togo. A standardized questionnaire was used to record socio-demographic data and sexual behavior patterns. A blood sample was taken and SD Bioline Duo VIH/Syphilis rapid test was used to test for HIV and syphilis.

**Results:**

A total of 2158 key populations (678 MSM, 1003 FSW and 477 DU), with an average age of 27.6 years (standard deviation 8.8 years) participated in the study. Prevalence of syphilis was 0.6% (95% CI = [0.3–1.0]) with no statistical significance between the three groups: null among MSM, 0.8% among FSW (95% CI = [0.37–1.63]) and 1.1% among DU (95% CI = [0.39–2.57]). There was no relation between HIV status and syphilis (*p* = 0.236). Among the 298 HIV-positive people, none was diagnosed with syphilis.

**Conclusions:**

Findings from this study reveal a low prevalence rate of syphilis among key populations in Togo. Specific interventions into HIV prevention programs should be reinforced to eliminate syphilis in Togo.

## Background

Syphilis is a sexually transmitted infection (STI) caused by *Treponema pallidum*. This chronic infection is highly contagious and it can be transmitted during sexual intercourse including vaginal, anal, or oral sex. At the early stage, syphilis causes ulcerative lesions which facilitates HIV transmission [1]. Among people living with HIV (PLHIV), it inflates the risk for cardiologic and neurological complications and treatment failure [2]. Despite the availability of inexpensive and effective treatment, syphilis remains a public health problem worldwide. According to the World Health Organization (WHO), in 2012, there were 18 million prevalent cases of syphilis worldwide, including 5.6 million newly infected cases among 15-to-49-year-olds [3].

In 2007, the WHO launched the global initiative to eliminate mother-to-child transmission of syphilis (congenital syphilis). In the recent years, 12 countries have been validated by the WHO as having eliminated mother-to-child transmission of syphilis and / or HIV. The estimates showed that the overall global burden of congenital syphilis decreased between 2012 and 2016, although non-significantly, from around 750,000 to 660,000 cases. Estimated adverse birth outcomes due to congenital syphilis decreased slightly from 397,000 to 355,000. Despite the decrease between 2012 and 2016, the number of affected women and infants remains unacceptably high [4].

Key populations, who are populations at higher risk for HIV such as female sex workers (FSW), men who have sex with men (MSM) and drug users (DU), including injection drug users (IDU), are disproportionally burdened by syphilis [5, 6]. In the United States where syphilis cases have rebounded since 2005 after being on the verge of elimination in 2000, about 80% of the increasing male primary and secondary syphilis cases were attributed to MSM [7, 8]. High prevalence of syphilis have also been reported among key populations in sub-Saharan Africa which bear the heaviest burden of HIV [9]. Several biological, behavioral and structural risk factors are associated with this high HIV rate among key populations: unprotected sex, presence of other STIs, lack of access to condoms, multiple concurrent sex partners (males and females for MSM), lack of access to health care and prevention services, physical and sexual violence, challenging legal and sociopolitical environment, poverty, stigmatization and discrimination [10–12]. To overcome the lack of access to health care and prevention services, rapid tests, which have proved to be cost effective and acceptable, are widely implemented to improve HIV and syphilis screening coverage among key populations [13].

In Togo, HIV prevalence among key populations ranges from 11 to 13% compared to 2.1% in the general population [14]. Data on the prevalence of syphilis are regularly obtained through a sentinel surveillance system which is designed to monitor trends of STI prevalence among pregnant women attending antenatal clinics. Based on this monitoring system, the prevalence of syphilis was estimated at 1.2% in 2011 [15]. This prevalence was approximately half of that reported the same year using a second-generation surveillance protocol among FSW and their clients with 2.2 and 2.3%, respectively [16]. No data on syphilis are available in other key populations, specifically among MSM who account for more than 80% of syphilis burden in developed countries [8, 17]. In 2017, we conducted a nationwide study to estimate the prevalence of syphilis among FSW, MSM and DU in Togo.

## Methods

### Study design, sampling and recruitment

This study was a bio-behavioral cross-sectional study conducted in August and September 2017 in eight towns of Togo. Prior to the study, locations specific to each group of key population were identified during preliminary visits with the help of leaders from these communities. MSM were recruited using a Respondent Driven Sampling (RDS) method. MSM community leaders were the first “seeds”. A total of 28 seeds were identified at first based on their roles in their community and on their representativeness. Each seed from the first wave selected had to represent at least one sub-group: passives, actives, bisexuals, gays, etc. Each participant was then given three coupons with a unique identification code to recruit three other seeds in their network until the required sample size for each group was reached. FSW and DU were recruited in “hot spots” and drug-dealing and consumption locations, respectively. Inclusion criteria for the three groups were being 18 years or older, living in Togo for a minimum of 3 months at the time of the study, and being in possession of a recruitment coupon. In addition to these criteria, criteria specific to MSM were having had anal sex with a man in the previous 12 months, for FSW having had sex in exchange for money as a compensation in the previous 12 months and for DU, consuming heroin, cocaine or hashish at the time of the study.

### Study procedures

After eligibility screening and informed consent approval, a structured and standardized questionnaire was administered by trained study staff during a face-to face-interview. Family Health International (FHI) 360 validated guide for bio-behavioral surveys was adapted to collect information on socio-demographic characteristics, risky sexual behaviors, STIs, HIV prevention methods, HIV testing history, access to health care services, and HIV knowledge. The standardized questionnaires were used across the three populations with slight adaptations depending on the population.

### Laboratory testing

Two HIV rapid tests were performed using SD Bioline Duo VIH/Syphilis rapid test kits which are endorsed by the WHO [18]. The WHO prequalification performance evaluation of this rapid diagnostic test reported a final sensitivity for HIV antibodies of 100, 95% confidence interval (95% CI) [98.2–100] and specificity of 99.5% [97.2–100] compared to the reference assays. All biological tests analyses were completed in the “Laboratoire de Biologie Moléculaire (BIOLIM)” (Molecular biology laboratory), Université de Lomé, which is the main HIV laboratory research unit in Togo.

### Statistical analysis

The sample size was calculated using a single proportion population formula with a 95% confidence level, 1% margin of error and 1% estimated prevalence of syphilis among FSW in Togo [16]. A 10% non-response rate was considered and the minimum number for each key population was estimated at 418. The required minimum sample size for comparing the three groups of key populations was estimated at 1254. Descriptive analyses were performed and results were presented with frequency tabulations and percentages. Prevalence rates were estimated with their 95% confidence interval. Fisher’s exact test was used to compare the relationship between categorical variables. All analyses were performed using R® version 3.4.3 software.

### Ethical consideration

This study was approved by the “Comité de Bioéthique pour la Recherche en Santé (CBRS)” (Bioethics Committee for Health Research) from the Ministry of Health of Togo (. Potential participants were told about the study purpose and procedures, potential risks and protections, and compensation. Informed consent was documented with signed consent forms prior to participation in the study.

## Results

### Socio-demographic and clinical characteristics

A total of 678 MSM, 477 DU and 1003 FSW, with an average age of 27.6 years, standard deviation of 8.8 years participated in the study. The majority of participants (*n* = 1154; 53.5%, *p* < 0.0001) had a secondary school education level and 342 (15.9%, *p* = 0.0027) were married. HIV prevalence was 13.8% across the three populations, with the highest prevalence among MSM (22.0%) (*p* < 0.001). Socio-demographic characteristics are summarized in Table 1.

**Table 1:** Socio-demographic characteristics of key populations in Togo in 2017 (*N* = 2158)

	MSM (*n* = 678) n (%)	FSW (*n* = 1003) n (%)	DU (*n* = 477) n (%)	Total (N = 2158) n (%)
Age (years)				
Mean ± standard deviation	24.1 ± 5.6	28.4 ± 9.1	31.2 ± 10.2	27.6 ± 8.8
Sex				
Female	–	1003 (100.0)	30 (6.3)	1003 (47.9)
Male	678 (100.0)	–	447 (93.7)	1125 (52.1)
Marital status				
Married	43 (6.3)	139 (13.9)	160 (33.5)	342 (15.8)
Not married	634 (93.5)	813 (81.1)	316 (66.3)	1763 (81.7)
MD	1 (0.2)	51 (5.0)	1 (0.2)	53 (2.45)
Education level				
No education	5 (0.7)	172 (17.2)	27 (5.7)	204 (9.5)
Primary school	56 (8.3)	287 (28.6)	143 (30.0)	486 (22.5)
Secondary school	375 (55.3)	494 (49.3)	285 (59.7)	1154 (53.5)
University	242 (35.7)	50 (4.9)	22 (4.6)	314 (14.5)
City				
Lomé (capital city)	466 (68.7)	571 (56.9)	240 (49.7)	1277 (59.2)
Other cities	212 (31.3)	432 (43.1)	237 (50.3)	881 (40.8)
HIV status				
HIV negative	529 (78.0)	871 (86.8)	460 (96.4)	1860 (86.2)
HIV positive	149 (22.0)	132 (13.2)	17 (3.6)	298 (13.8)

MD = Missing data, DU: Drugs users, FSW: Female sex workers, MSM: Men who have sex with men

### Prevalence of syphilis infection

A total of 13 participants were diagnosed with syphilis yielding a prevalence of 0.6, 95% CI [0.3–1.0]. Table 2 summarizes the prevalence of syphilis infection according to key population and sociodemographic characteristics.

**Table 2:** Prevalence of syphilis infection among key populations in Togo, 2017

	N	n	Prevalence (%)	95% CI	*p* value
Age (years)					0.001
< 30	1474	3	0.2	[0.1–0.7]	
≥ 30	684	10	1.5	[0.7–2.8]	
Sex					0.035
Male	1125	3	0.3	[0.1–0.9]	
Female	1033	10	1.0	[0.5–1.8]	
City					0.014
Lomé (capital city)	1277	12	0.9	[0.5–1.7]	
Other cities	881	1	0.1	[0.0–0.7]	
Key populations					0.015
MSM	678	0	0.0	[0.0–0.5]-	
FSW	1003	8	0.8	[0.4–1.6]	
DU	477	5	1.1	[0.4–2.6]	
HIV status					0.236
HIV negative	1860	13	0.7	[0.4–1.2]	
HIV positive	298	0	0.0	[0.0–1.2]	

CI: Confidence interval

DU: Drugs users, FSW: Female sex workers, MSM: Men who have sex with men

The prevalence of syphilis was 0.8, 95%CI [0.4–1.6%] among FSW, 1.1, 95%CI [0.4–2.6]) among drugs users and 0, 95% CI [0–0.5%] among MSM (*p* = 0.015).

Among participants infected with syphilis, three were aged less than 30 (0.2%; 95% CI = [0.1–0.7], *p* = 0.001). Most of them were females (*n* = 10, 1.0%; 95% CI = [0.5–1.8], *p* = 0.035) and lived in the capital city of Lomé (n = 10, 0.9%; 95% CI = [0.5–1.7], *p* = 0.014).

There was no relation between HIV status and syphilis. All the 13 participants who were infected with syphilis were HIV negative (*p* = 0.236).

## Discussion

This study evaluating the prevalence of syphilis among 2158 key populations including MSM, DU and FSW in a national survey in Togo showed a low prevalence of syphilis estimated at 0.6%, with no association with HIV infection.

Among FSW, our study reported a prevalence of 0.8%, which represents a decrease in syphilis infection among this population from previous estimations. A study conducted in 2011 in Togo among 1106 FSW reported a prevalence of 2.2% and being 30 years and older (odds ratio (OR) =5.03; 95% CI [1.95–13.49]), being married or living with a partner (OR = 3.11; CI 95% [1.16–8.83]), being an undeclared or secret FSW (OR = 3.89; CI 95% [1.60–9.54]) and HIV infection (OR = 3.41; IC 95% [1.53–7.41]) were factors associated with syphilis infection [16]. In Burkina Faso, a neighboring country of Togo, a study was conducted between 2013 and 2014, with the inclusion of 1045 FSW. The estimated prevalence of active syphilis ranged from 0.0 to 2.2%, which are quite comparable to our results. Factors associated with the presence of syphilis marker were low education and high number of clients [19].

Among MSM, the syphilis prevalence reported was null. Similar findings were observed among MSM in the West African region. In the CohMSM study conducted in 2016 in four capital cities of West Africa (Abidjan, Bamako, Lomé, Ouagadougou), the overall prevalence was 1.3% among 524 MSM enrolled [20]. In Lomé, only one case (0.8%) was reported among 124 MSM enrolled [21]. This trend of low prevalence of syphilis was also reported in East Africa. One study in Dodoma (Tanzania) reported a prevalence of syphilis of 0.2% among 409 MSM and in another one carried out in Dar-Es-Salaam and Tanga (Tanzania), the syphilis prevalence was 2.5 and 0.0%, respectively [22, 23].

In our study, among 477 DU, including 30% of IDU, the prevalence of syphilis was 1.1%. In a survey conducted in Ethiopia in 2015, the prevalence of syphilis was 5.1% among the 237 participants with 40% of those injecting drugs [24]. In another study conducted in Lagos, Nigeria, the prevalence of syphilis among 328 male injection drug users was 1.9%, which is approximately similar to our results in the same West African context [25].

Overall, these data demonstrate a general decreasing trend in syphilis prevalence in sub-Saharan Africa among key populations and confirm a very low circulation of syphilis in Togo among key populations. Similar observations were reported in South Africa. In this country, in 2017, using the Spectrum-Sexually Transmitted Infection model among adults (15–49 years old), the estimated female and male prevalence of syphilis were 0.50% (95% CI: 0.32–0.80%) and 0.97% (0.19–2.28%), respectively. Between 1990 and 2017 the estimated prevalence of syphilis declined steadily in women and men, probably in part reflecting improved treatment coverage [26]. The reasons for this low prevalence in Togo are unclear. However, the systematic use of antibiotics in case of genital infection could be an explanation. Further surveillance, epidemiological and comparative studies are needed to assess whether there is indeed a difference in syphilis prevalence rates in SSA and other parts of the World, and to identify specific interventions or hypotheses that could explain this fact.

Strengths of this study include the large sample size of the three main types of key populations, which is among the firsts of the sort in Togo and in West Africa. To our knowledge, this is the first study comparing prevalence of syphilis in the three main key populations. The majority of studies focused on one specific population such as MSM [22, 23], FSW [16] or DU [25]. In addition, we used SD Bioline HIV/Syphilis Duo, a rapid test that simultaneously detects antibodies to HIV and syphilis, and has the potential to further benefit clinics and patients by reducing costs, testing complexity, and patient wait times. SD DUO syphilis sensitivity and specificity, when compared to rapid plasma reagin, were 85.7 and 96.8%, respectively, and 69.7 and 99.7%, respectively, when compared to *Treponema pallidum* particle agglutination (TPPA) [27, 28]. However, there were few limitations to this study including the fact that factors associated with syphilis infection were not evaluated because of low prevalence of syphilis in this population. Another limitation is that the SD Bioline HIV/Syphilis Duo test detects only treponemal antibodies and does not allow for the distinction between past and current infection. Also, a confirmatory test with non-treponemal test was not performed because the WHO recommends a second test (PRP non treponemal test) only in countries with high prevalence of syphilis (> 5%). Finally, syphilis diagnosis did not include a clinical assessment. Nevertheless, these limitations are minimized by the cross-sectional study design, and the use of standardized data collection instruments and laboratory measurements, as well as the large sample size.

## Conclusion

Our data suggest that syphilis infection is low among key populations in Togo with an estimated of 0.6%. There is a need to confirm these results in these populations and in other populations such as pregnant women to confirm the elimination of syphilis in Togo. Active surveillance and systematic reporting are therefore needed. Integration of syphilis with HIV testing may facilitate the implementation of this screening strategy in settings where individuals have increased sexual risk.

## Abbreviations

95%CI: 95% Confidence Interval
DU: Drug users
FHI: Family Health International
FSW: Female Sex Workers
IDU: injection drug users (IDU)
MSM: Men who have sex with Men
OR: Odds Ratio
PLHIV: People Living with HIV
RDS: Respondent Driven Sampling
SSA: Sub-Saharan Africa
STI: Sexually Transmitted Infection
TPPA: Treponema pallidum particle agglutination
WHO: World Health Organization
